# Effects of *Bacillus subtilis* T6-1 on the Rhizosphere Microbial Community Structure of Continuous Cropping Poplar

**DOI:** 10.3390/biology11050791

**Published:** 2022-05-23

**Authors:** Junkang Sui, Qianqian Yu, Kai Yang, Jiayi Yang, Chenyu Li, Xunli Liu

**Affiliations:** 1 College of Agronomy and Agricultural Engineering, Liaocheng University, Liaocheng 252000, China; yjy2002292022@163.com (J.Y.); lichenyu200209@163.com (C.L.); 2 Department of Architecture and Civil Engineering, Shandong Liaocheng Vocational and Technical College, Liaocheng 252000, China; yuqianqian0227@163.com; 3 Liaocheng Food and Drug Inspection and Testing Center, Liaocheng 252000, China; lcjcncpk@lc.shandong.cn; 4 College of Forestry, Shandong Agricultural University, Tai’an 271000, China; xunliliu@163.com

**Keywords:** poplar, rhizosphere, continuous cropping obstacles, *Bacillus subtilis*, microbiology community structure

## Abstract

**Simple Summary:**

Long-term continuous cropping of poplar has led to a decline in soil fertility, the timber yield of poplar has decreased, and diseases and pests have increased. We aimed to develop a biological agent that regulates the structure of the microbial community of the poplar rhizosphere and alleviates the effects of continuous poplar cropping. The research showed that T6-1 could improve the structure of the microbial community of the poplar rhizosphere and promote the growth of poplars. Thus, it could be used as a biological control agent against continuous cropping obstacles for poplar trees.

**Abstract:**

The continuous cropping obstacles in poplar cultivation cause declines in wood yield and serious soil-borne diseases, mainly because of structural alterations in the microbial community and the aggregation of pathogenic fungi. *Bacillus subtilis* T6-1, isolated from poplar rhizospheric soil, has strong antagonistic effects on poplar pathogens. We aimed to investigate the effects of *B. subtilis* T6-1 on the structure of the microbial community in the poplar rhizosphere. Poplar seedlings were replanted in three successive generations of soil. The diameter at breast height, plant height, and the number of culturable bacteria of the poplars inoculated with T6-1 exceeded those in the non-inoculated control group. qPCR analysis revealed that the total abundance of T6-1 bacteria in the treated poplars was remarkably higher in contrast to that in the control group. Illumina MiSeq sequencing was employed to track the alterations in diversity and structure of the total microbial community in the poplar rhizosphere inoculated with *B. subtilis* T6-1. Fungal diversity and abundance in the T6-1 rhizosphere were remarkably lower in contrast with those in the control rhizosphere. The proportion of *Bacillus* sp. in the total bacterial community in the T6-1 and control groups was 3.04% and 2.38%, respectively, while those of the *Rhizoctonia* sp. was 2.02% and 5.82%, respectively. In conclusion, *B. subtilis* T6-1 has the potential to serve as a microbial agent, enhancing the structure of the rhizosphere microbial community as well as promoting tree growth in poplar cultivation.

## 1. Introduction

Poplar is the main tree species used for directional cultivation, wood processing, and timber production. Poplar plantations account for 13.5% of China’s total planted forest area [1,2]. Even though the contribution of poplar plantations to the global timber supply is limited to date, poplar plantation areas have been increasing rapidly [3]. However, in recent decades, because of economic development and the increasing population, the demand for wood has greatly increased, whereas the land that can be used for afforestation is limited. Therefore, only short-rotation and continuous cropping can be applied in plantation forests [4]. Research on the growth of seedlings of different generations in continuous poplar plantations showed that with an increase in continuous cropping generation, plant height, biomass, as well as diameter at breast height (DBH) decreased remarkably, and growth was obviously inhibited [5]. In contrast with the first generation, the average height of second- and third-generation seedlings was decreased by 4.30% and 20.34%, respectively, the average DBH was decreased by 6.55% and 20.83%, respectively, and the biomass per plant was decreased by 5.66% and 34.46% [6].

Continuous cropping obstacles have been analyzed from the perspectives of alterations in the physical and chemical properties of soil, self-allelopathy, and the deterioration of the soil’s biological environment [7,8]. Prolonged continuous cropping reportedly leads to alterations in the soil environment, which affect plant growth in the region [9,10]. At the same time, the number of microorganisms changes, and the community structure deteriorates [11]. Such alterations in soil microbial community diversity and structure can partly explain the vulnerability of continuous cropping farmland soil [12,13]. The soil microbial community, especially rhizospheric microorganisms, can impact plant growth via feedback mechanisms. Lu et al. [14] reported that the flowering time of *Arabidopsis thaliana* may be delayed by rhizosphere microbes, thus stimulating further plant growth. Yuan et al. [15] suggested that plants attract beneficial rhizosphere communities as a response to the exposure of aboveground parts to pathogens, which is conducive to plant growth. Tan et al. [16] suggested *Mycocentrospora* as a potential pathogen that accounts for the continuous cropping of *Panax notoginseng* obstacles.

*Bacillus* species are progressively being utilized in agriculture to enhance plant growth, as well as to protect plants against pathogens [17]. *Bacillus subtilis* PTS-394 can support plant growth and impede soil-borne diseases in tomato [18]. *Bacillus* spp. are among the most effective biocontrol agents as an alternative to synthetic fungicides [19]. Certain strains of *B. subtilis* are effective biocontrol agents against *Fusarium oxysporum* and are marketed as biopesticides [20]. The complex interactions between inoculated *B. subtilis* strains and indigenous microbial communities in the rhizosphere must be carefully studied because *B. subtilis* can produce high levels of secondary metabolites, which might affect indigenous microorganisms [21]. Cai et al. [22] reported that bioorganic fertilizer sustains a more stable soil microbiome relative to chemical fertilizer for monocropping.

The impact of inoculants on the microbial community of the native rhizosphere has been considered to be one of the factors that determine the success of biological control [23]. *B. subtilis* T6-1, isolated from poplar rhizosphere soil, has strong antagonistic effects against the poplar rot pathogens *F. oxysporum*, *Fusarium solani*, *Rhizoctonia solani*, *Alternaria alternata*, and *Phytophthora capsici*.

Herein, we explored the bacterial along with the fungal community structures in rhizospheres of continuously cropped poplars and assessed the effects of the fungicidal strain T6-1 on these communities. Based on both the DBH of the poplar trees and the biomass of bacteria, as well as fungi, we explored the feasibility of using T6-1 in relieving continuous cropping obstacles and improving the structural element of bacterial communities.

## 2. Materials and Methods

### 2.1. Study Site and Experimental Design

The study site (31°56′ N, 117°08′ E) is located in Taian City in Shandong Province, in eastern China. The study site is located close to Dawen River, however, it has not experienced flooding in the past several years. This region is typified by a growing season averaging 195 frost-free days and a temperate monsoon climate. Its average annual temperature is 12.9 °C, (with a range from −20.7 °C to 38.1 °C). It experiences annual precipitation of approximately 697 mm, in the period between July and September.

We set up the experiment in an artificial forest in an area of about 3.5 km^2^. Every treatment area runs nearly south to north, with a rectangular shape of 12 m by 80 m. The study in the artificial forest had a block design. Every treatment area consisted of three parallel rows, with two-meter apart planted seedlings in rows 4 m apart. The T6-1 group and the control (CK) group were 20 m apart. 

The planting of the *Populus × euramericana* cv. ‘Neva’ seedlings at the site was conducted for three generations. After the 3 generations of continuous cropping without rotation, seedlings of similar height were replanted on 30 March 2018. The seedlings were inoculated thrice with microorganism zymotic fluid containing T6-1 at a level of 6.97 × 10^8^ cfu/mL. The T6-1 fermentation medium consisted of 20 g/L of glucose, 20 g/L of corn flour, 4 g/L of corn syrup, 7.5 g/L of (NH_4_)_2_SO_4_, 3 g/L CaCO_3_, and distilled H_2_O, up to one liter. In the first treatment, we dipped the roots in the medium for five minutes. The fermentation medium was diluted ten-fold with irrigation water, and then 1.39 × 10^10^ cfu (1.39 × 10^6^ cfu/g soil) bacteria were applied per seedling. Immediately after planting, we irrigated the roots with the diluted medium (200 mL/seedling). Finally, 4 L of water with the diluted medium was used to irrigate each seedling. The CK group was treated using sterilized zymotic fluid in the same approach. Each treatment consisted of about 60 seedlings. We measured the DBH (1.3 m above the level of the ground) of all seedlings in October 2018 as well as October 2020.

### 2.2. Sample Collection and Count of Culturable Microorganism

The collection of the samples was conducted on 30 October 2020. From the north, south, and central locations, five trees were selected in a random manner, and the roots were sampled. We shook off the excess bulk soil and treated the soil adhering to the roots as the rhizosphere soil [24]. The soil samples collected in the south, central, as well as north locations at a depth of 15–20 cm were mixed to generate three triplicate soil samples from each location for each treatment. The sampled soils were put in individual sterile plastic bags and then put in ice for transportation to our laboratory and kept at −80 °C until DNA isolation. Pooling of replicate samples was conducted, then a quartile approach was employed to determine the final samples for analysis (T6-1-1, T6-1-2, T6-1-3, and CK1, CK2, CK3).

Serial dilution of the soil samples was conducted up to 10^−6^-fold, and the resulting dilutions were inoculated in nutrient agar medium for counting bacteria or on Martin agar medium constituted with 30 g/mL of streptomycin for counting fungi. The incubation of the agar plates was performed under 28 ± 2 °C conditions for 2–3 days [25].

### 2.3. Determination of Edaphic Properties

The soil OC (organic carbon) was measured using the Walkley–Black approach [26]. Ten milliliters of 1 N potassium dichromate were mixed with 20 mL of H_2_SO_4_ (concentrated), introduced to 0.1 g sieved and dried soil, followed by gentle mixing via rotation for one minute. The mixture was incubated for ten minutes at 150 °C and cooled to room temperature. After that, we diluted the samples to 200 mL using deionized H_2_O and 10 mL of H_3_PO_4_, 0.2 g NH_4_F and we added 10 drops of (C_6_H_5_)_2_NH indicator. After that, the titration of the excess dichromate was performed with Morh salt solution (0.5 N FeNH_4_SO_4_ and 0.1 N H_2_SO_4_). The available P (AP) was assessed by the molybdenum blue approach [27], using a spectrophotometer (UV2550, Shimadzu, Japan). The available K (AK) was assessed after extracting the soil with C_2_H_7_NO_2_. The automatic Kjeldahl distillation-titration unit was employed to quantify the Total N (TN) (Foss, Denmark, Sweden). The pH of the soil samples was determined with a pH meter (Mettler Toledo, Zurich, Switzerland).

### 2.4. DNA Isolation and PCR Amplification

The isolation of microbial DNA of the two groups was performed using an E.Z.N.A. Soil DNA Kit (Omega Bio-Tek, Norcross, GA, USA) was applied. The V3–V4 bacterial 16S ribosomal RNA region was subjected to the PCR assay (95 °C for three minutes; and subsequently by 27 cycles of 95 °C for 30 s, 55 °C for 30 s, and 72 °C for 45 s; and a final extension at 72 °C for ten minutes) using the following primers: 338F, 5′-barcode-ACTCCTACGGGAGGCAGCA-3′and 806R, 5′-GGACTACHVGGGTWTCTAAT-3′ [28], where the barcode is an eight-nucleotide sequence unique to every sample. PCR was conducted in triplicate in 20-μL reaction mixtures of 5× FastPfu Buffer (4 μL), 2.5 mM dNTPs (2 μL), each primer (5 μM; 0.8 μL), FastPfu Polymerase (0.4 μL), 10 ng template DNA, and ddH_2_O to a final 20 µL volume.

The fungal ITS1 PCR conditions were 95 °C for 3 min; and subsequent 35 cycles at 95 °C for 30 s, 55 °C for 30 s, and 72 °C for 45 s; and a final extension at 72 °C for 10 min) using the following primers: ITS1F, 5′-barcode-CTTGGTCATTTAGAGGAAGTAA-3′ and 2043R, 5′-GCTGCGTTCTTCATCGATGC-3′ [29], where the barcode is an eight-nucleotide sequence unique to every sample. PCR was carried out in triplicate in 20-μL reaction mixtures, as described above.

### 2.5. Real-Time (q)PCR

The oligonucleotide primers employed to amplify the bacterial 16S ribosomal RNA V3–V4 region along with the fungal ITS1 region are shown in Section 2.3. The qPCR reaction mixture contained 25-μL of 2× SYBR Green qPCR Master Mix (12.5 μL), each primer (10 μM; 0.5 μL), ddH2O (9.5 μL), and template DNA (2 μL). The amplification parameters were: 95 °C for 10 min, 40 cycles of 95 °C for 15 s and 60 °C for 1 min, and melting-curve analysis. The baseline start was set at 3, and the baseline end at 11 to 12. The data were analyzed using the ABI 7500 fluorescence quantitative analyzer (Thermo Fisher Scientific, Waltham, MA, USA).

### 2.6. Illumina MiSeq Sequencing

The purification of the amplicons from 2% agarose gels was performed with AxyPrep DNA Gel Extraction Kit (Axygen Biosciences, Union City, CA, USA), as described by the manufacturer. After that, the QuantiFluor-ST Kit (Promega, Madison, WI, USA) was employed to quantify the products. Thereafter, we pooled the amplicons at equimolar amounts for paired-end sequencing (2 × 250) on an Illumina MiSeq platform as described by the manufacturer. Generated raw reads are available in the NCBI Sequence Read Archive database (SRR5901141).

### 2.7. Processing of Sequencing Data

The uchime function in the Mothur software was used to remove chimera sequences and obtain high-quality sequences for further data analysis. De-multiplexing of the raw fastq files was conducted, followed by quality-filtering with QIIME (version 1.9.1, Center for Applied Microbiome Science, Pathogen and Microbiome Institute, Northern Arizona University, Flagstaff, AZ, USA) as follows: (i) A sliding window of 50 bp was set if the average mass value in the window was <20, all the sequences of the back end of the base from the front end of the window were cut off, and sequences whose length was <50 bp after quality control were removed; (ii) The overlap sequence was spliced according to overlap base overlap. The maximum mismatch rate of overlap was 0.2, and the length of overlap was >10 bp. The sequences that could not be stitched together were removed; (iii) The sequences were separated into each sample, per the barcode, as well as primers at both ends of the sequence. The sequences with ambiguous bases were removed (two base mismatches were allowed for primers) [30].

The clustering of the OTUs (operational taxonomic units) was performed with a 97% similarity cutoff using UPARSE (version 7.1, http://drive5.com/uparse/, accessed on 20 September 2021) [31,32], and UCHIME was applied to determine and eliminate chimeric sequences [33]. The RDP Classifier (http://rdp.cme.msu.edu/, accessed on 20 September 2021) was employed to analyze the taxonomy of every 16S rRNA gene against the Silva (SSU128) 16S rRNA data resource, with a 70% confidence threshold [34]. The RDP Classifier was utilized to assess the taxonomy of every ITS sequence against the UNITE 7.0/ITS data resource [35] with a 70%confidence threshold.

### 2.8. Statistical Analyses

All of the results were given as the mean ± standard deviation (SD). Remarkable differences in the quantities of culturable, as well as total microorganisms, DBH of poplar trees, plant height, and diversity along with the richness indices between the T6-1 and CK groups were identified using one-way ANOVA followed by Duncan’s multiple ranger tests at a significance level of *p <* 0.05. All of the statistical analyses were implemented in SAS, version 9 (SAS Institute Inc., Cary, NC, USA).

## 3. Results

### 3.1. Changes in Poplar Tree DBH and Plant Height

In October 2018, after 6 months of growth, the poplars exposed to strain T6-1 were 9.40% thicker and 4.01% higher in contrast with non-inoculated CK trees. In October 2020, the poplars exposed to the T6-1 strain were 11.72% thicker and 5.68% higher in contrast with those in the CK group. The DBH increased by 85.62 mm between October 2018 and October 2020 in the T6-1 group and by 75.90 mm in the CK group (Table 1). Plant height increased by 7.19 m in the T6-1 group and by 6.74 m in the CK group. These findings indicated that the application of strain T6-1 promoted the growth of the poplars.

### 3.2. Changes in Edaphic Properties from March 2018 to October 2020

Soil OC, AP, AK, TN, and pH were not remarkably different prior to treatment with T6-1 zymotic fluid or sterile zymotic fluid (CK) in March 2018 (Table 2). The OC upon T6-1 treatment 0.85 (g/kg) was dramatically higher in contrast to that in CK plots in October 2020. Soil AP, AK, and TN were 11.34 (mg/kg), 92.56 (mg/kg), and 300.91 (mg/kg), respectively, which was dramatically higher in contrast to those in the CK plots in October 2020. The pH was substantially increased in the soils exposed to the T6-1 strain. Soil OC and AK in the CK plots were substantially higher in October 2020 relative to that in March 2018. However, AP and AK were not dramatically different between March 2018 and October 2020 in CK plots.

### 3.3. Differences in Microbequantities in Rhizosphere Soils of Poplars

The culturable bacteria number in the rhizosphere soil escalated dramatically after the T6-1 bacterial zymotic fluid treatment in contrast to CK. The number of culturable bacteria in the T6-1 rhizosphere was ten times higher in contrast to that in the CK rhizosphere. However, the number of culturable fungi in the T6-1 rhizosphere was 54.05% relative to that of the CK rhizosphere (Table 3). The qPCR results illustrated that the total amount of bacteria in the T6-1 rhizosphere was 64.79% higher in contrast to that in the CK rhizosphere, while the total amount of fungi was 25.72% lower in contrast to that in the CK rhizosphere (Table 3).

### 3.4. Estimators of the Diversity along with Species Richness of Microbial Communities

**Figure 1:**
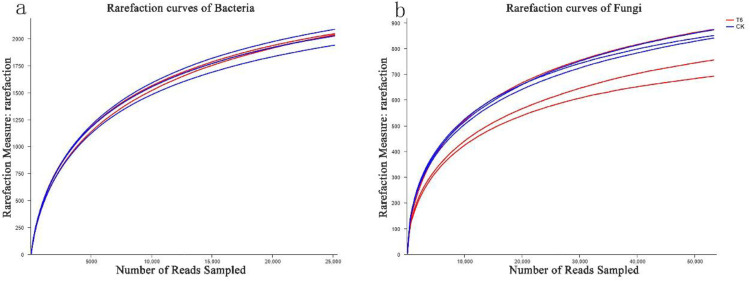
Rare-faction curves of (**a**) communities of bacteria and (**b**) fungi. The rare-faction curves exhibiting influences of a dissimilarity cut-off of 3% on OTUs numbers uncovered in the study groups. The T6-1 group consisted of poplar trees, which were exposed to bacterial zymotic fluid with T6-1. The CK group consisted of poplar trees, which were exposed to an equivalent volume of sterile zymotic fluid. We analyzed three replicates for every treatment.

**Figure 2:**
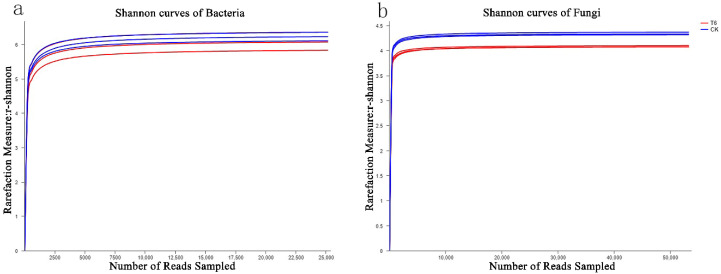
Shannon curves of communities of (**a**) bacteria and (**b**) fungi. Shannon curves, illustrating the influences of a dissimilarity cut-off of 3% on OTUs numbers uncovered in study groups. The T6-1 group consisted of poplar trees, which were exposed to bacterial zymotic fluid with T6-1. The CK group consisted of poplar trees, which were exposed to an equivalent volume of sterile zymotic fluid. We analyzed three replicates for every treatment.

We filtered the sequencing data to exclude low-quality and short reads, yielding 52,807 and 50,480 bacterial 16S rDNA and 76,609 and 68,753 fungal ITS sequences in the T6-1 and CK groups, respectively. With a clustering dissimilarity cut-off of 3%, the reads were classified into diverse OTUs. The 0.03 distance level rarefaction curves of the bacterial as well as fungal communities did not reach an asymptote (Figure 1). Hence, the sequencing data did not fully represent all of the communities present in the samples. However, by integrating the Shannon diversity index with the rarefaction curves (Figure 2), we established that, with an increasing number of reads, the Shannon diversity curves approached a plateau. Hence, adequate data were obtained to facilitate the examination of the communities.

The diversity along with the richness indices in the soil samples were analyzed (Table 4). Notably, T6-1 and CK groups showed similar bacterial richness and diversity. ACE (abundance-based coverage estimator) along with Chao values, which are species richness indicators, were relatively higher for the rhizosphere bacterial community in the CK in contrast to that in the T6-1 group, whereas OTU values were slightly lower in CK relative to that in T6-1. The Shannon along with Simpson diversity index values exhibited a similar trend, with lower diversity index values being linked to larger Simpson values.

As for fungi, ACE and Chao values in the T6-1 group were dramatically lower relative to those in the CK. In addition, the Shannon diversity index was remarkably lower in the T6-1 relative to that in the CK group. No remarkable differences were reported in the Simpson index, even though the values were relatively higher in the T6-1 group. These data illustrated that the CK group had a richer and more diverse fungal community relative to the T6-1 group. The only negligible differences were reported in the diversity as well as species richness of the bacterial communities between T6-1 and CK.

### 3.5. Effects of T6-1 on Community Composition and Structure

**Figure 3:**
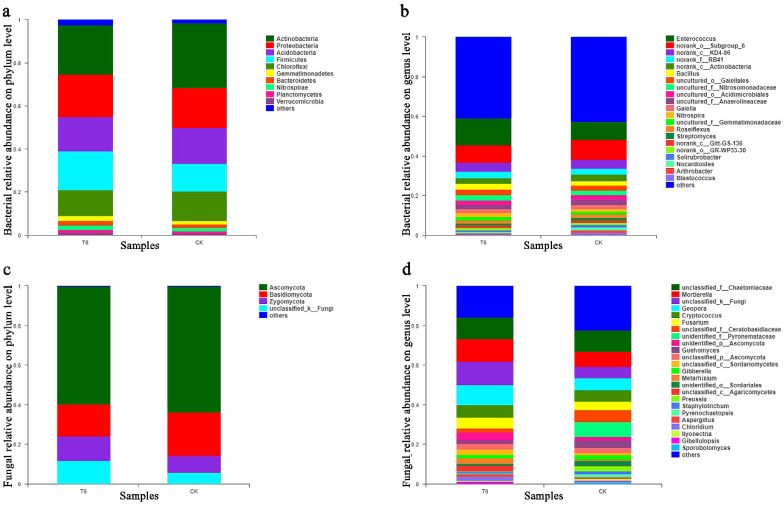
Structures of communities of bacteria and fungi in study groups (**a**) Phylum level bacterial relative abundance (**b**) Genus level bacterial relative abundance (**c**) Phylum level fungal relative abundance (**d**) Genus level fungal relative abundance. Stacked bar graphs illustrating major genera relative abundances. The T6-1 group consisted of poplar trees, which were exposed to bacterial zymotic fluid with T6-1. The CK group consisted of poplar trees, which were exposed to an equivalent volume of sterile zymotic fluid. We analyzed three replicates for every treatment. The non-metric multidimensional scaling (NMDS) plot on OTU level could be found in Supplementary Figure S1.

**Figure 4:**
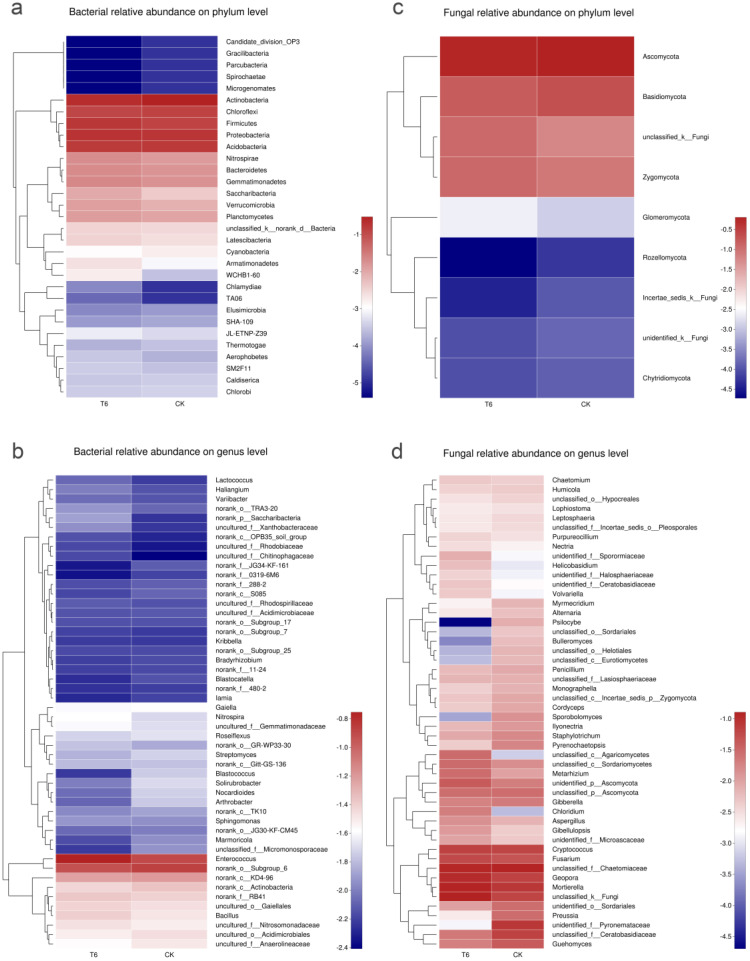
Bacterial and fungal distribution hierarchical clustering heatmaps (**a**) Phylum level bacterial relative abundance (**b**) Genus level bacterial relative abundance (**c**) Phylum level fungal relative abundance. (**d**) Genus level fungal relative abundance. The heatmaps illustrating distribution of diverse communities from the two groups at the genus level. The T6-1 group consisted of poplar trees, which were exposed to bacterial zymotic fluid containing T6-1. The CK group consisted of poplar trees, which were exposed to an equivalent volume of sterile zymotic fluid. We analyzed three replicates for every treatment.

The Mothur program was employed to classify the sequences. The dominant bacterial phyla were Actinobacteria (22.90% and 30.21% in T6-1 and CK groups, respectively), Proteobacteria (19.64% and 18.29% in T6-1 and CK groups, respectively), and Acidobacteria (16.04% and 16.70% in the T6-1 and CK groups, respectively). The dominant fungal phyla were Ascomycota (59.41% and 63.76% in the T6-1 and CK groups, respectively) and Basidiomycota (16.28% and 21.78% in the T6-1 and CK groups, respectively) (Figure 3). Heatmap-based analysis of the community structure confirmed that Actinobacteria, Proteobacteria, as well as Acidobacteria were the predominant bacterial phyla. On the other hand, Ascomycota, along with Basidiomycota, were the predominant fungal phyla (Figure 4a,c).

A similar composition was found in the overall bacteria of the two groups, while the distribution of each genus differed (Figure 3). *Enterococcus* accounted for 13.38% of total classifiable bacterial sequences in the T6-1 group, however, for only 9.27% in the CK group (Figure 3b). The proportion of *Bacillus* were 3.04% and 2.38% in the T6-1 and the CK group, respectively. *Nocardioides* proportion in the T6-1 group was 0.63%, which was lower relative to that (1.37%) reported in the CK group. Similarly, the *Blastococcus* fraction in the T6-1 group was 0.48%, which was lower relative to that (1.47%) in the CK group, although this difference was not remarkable (*p* = 0.1455). The *Arthrobacter* proportion in the T6-1 group was 0.92%, which was remarkably lower relative to that (1.69%) in the CK group. The fraction of *Haliangium* in the T6-1 group was 0.76%, which was remarkably higher relative to that (0.53%) in the CK group. The fraction of *Mycobacterium* in the T6-1 group was 0.27%, which was remarkably higher relative to that (0.39%) in the CK group. The fraction of *Aeromicrobium* in the T6-1 group was 0.18%, which was remarkably higher relative to that (0.43%) in the CK group. On the contrary, the fraction of *Steroidobacter* in the T6-1 group was 0.39%, which was remarkably higher relative to that (0.26%) in the CK group. The heatmap also revealed the respective quantitative associations among fungi and bacteria in the T6-1 and CK groups. The fraction of *Enterococcus* was 15.11% and 10.6% in the T6-1 and CK groups, respectively, depicting the largest difference in bacteria between both groups. On the contrary, the proportion of *Blastococcus* was 0.54% and 1.69%, that of *Bacillus* sp. was 3.44% and 2.74%, while that of *Anaerobic rosaceae* was 2.34% and 2.72%, in the T6-1 and CK groups, respectively (Figure 4b).

Ascomycota accounted for 14.10% of total classifiable fungal sequences in the T6-1 group but only for 8.44% in the CK group (Figure 3d). The proportion of *Geopora* was 9.99% and 5.99% in the T6-1 and CK groups, respectively, while the fraction of *Fusarium* was 4.31% in the T6-1 group, which was relatively higher relative to that (4.03%) in the CK group. The proportion of *Rhizoctonia* was 2.02% and 5.82% in the T6-1 and the CK group, respectively. *Fusarium* and *Rhizoctonia* are causative agents of numerous diseases in plants. The fraction of *Cryptococcus* in the T6-1 group was 6.30%, which was higher relative to that (4.93%) in the CK group. Moreover, the fraction of *Preussia* in the T6-1 group was 0.98%, which was lower relative to that (2.72%) in the CK group. Regarding fungi, the proportion of *Geopora* in T6-1 and CK was 10.19% and 6.17%, respectively, based on heatmap analysis. The proportion of *Fusarium* species were 4.39% and 4.15%, while those of *Rhizoctonia* was 2.06% and 6.00% in the T6-1 and CK groups, respectively (Figure 4d).

**Figure 5:**
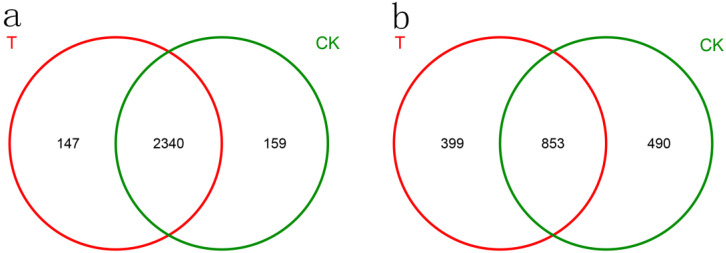
Venn diagram exhibiting unique, as well as shared (**a**) bacterial OTUs (**b**) fungal OTUs for the two treatments. The T6-1 group included poplar trees, which were exposed to bacterial zymotic fluid with T6-1. The CK group consisted of poplar trees, which were exposed to an equivalent volume of sterile zymotic fluid. We analyzed three replicates for every treatment.

Bacterial along with fungal OTUs frequent in both study groups are depicted in the Venn diagram in Figure 5. The number of bacterial OTUs reported for every treatment was as follows: 2487 for T6-1, and 2499 for CK, at 3% dissimilarity. The T6-1 group and the CK shared 2340 OTUs. Three primary genera were frequent in both groups: *Enterococcus*, *Bacillus*, as well as *Actinobacteria*. *Neochlamydia* and *Shimazuella* occurred only in the T6-1 group. *Rickettsiella*, *Paenisporosarcina*, and *Phytomonospora* occurred only in the CK group.

We uncovered 1252 fungal OTUs in the T6-1 group and 1343 in the CK group, at a 3% dissimilarity. T6-1 and CK shared 853 OTUs. *Mortierella*, *Geopora*, *Cryptococcus*, *Fusarium*, and *Guehomyces* were common to both groups. *Wilcoxina*, *Paraglomus*, *Corallomycetela*, and *Spirosphaera* were only present in the T6-1 group, while *Psilocybe*, *Gyrothrix*, *Chaetomella*, and *Articulospora* occurred only in the CK group.

## 4. Discussion

In studies on continuous cropping obstacles, physicochemical properties and autotoxic effects of continuous cropping soils are usually assessed, and causes of soil degradation are analyzed according to their effects on the physiological state of the forest land. Prolonged continuous cropping leads to changes in the soil microbial environment, which subsequently affect plant growth in the region [9,36]. Meanwhile, the number of microorganisms will change, and the community structure will deteriorate [11]. Imbalances in the soil microbial community present a major continuous cropping obstacle [37]. For a long time, continuous cropping obstacles caused the number of soil fungi to increase and the number of bacteria to decrease [38].

The DBH data of the poplar trees 16 months after the treatments revealed that the DBH of the T6-1 treatment group was increased by 13.03 mm on average when in contrast with the control group, and plant height increased by 7.19 m in the T6-1 group and by 6.74 m in the CK group, indicating an increase in wood yield. Based on these data, we conclude that T6-1 can promote poplar production. Similarly, inoculation with *B. subtilis* BEB-13bs escalated the yield per plant, as well as marketable yield along with fruit weight and length in tomato [39].

When the T6-1 strain was applied in the rhizosphere of poplars, the culturable and total amounts of bacteria were remarkably increased, whereas the number of fungi was dramatically decreased. In addition, OC, AP, AK, and TN were obviously increased upon treatment with T6-1 (Table 2). Thus, T6-1 application can improve low-fertility-type soil dominated by fungi, which is consistent with a previous report that suggested that when the density of beneficial microbial populations in the soil decreases and the number of pathogenic microorganisms increases, the rhizosphere soil microflora shifts from high-fertility “bacterial type” to low-fertility “fungal type” [40,41,42]. Janvier, Villeneuve, Alabouvette, Edel-Hermann, Mateille and Steinberg [43] suggested that prolonged continuous cropping could lead to a decrease in microbial community diversity and an increase in harmful microbial populations in soils. In our study, the fraction of *Rhizoctonia*, which is an important pathogenic fungus of poplar, was higher in the CK relative to that in the T6-1 plots.

While bacterial diversity did not change upon T6-1 application, fungal diversity did increase in our study. Actinobacteria, Proteobacteria, and Acidobacteria were the dominant phyla. Actinomycetes are important soil bacteria, and various studies have indicated they are antagonistic microorganisms [44,45,46]. Members of Acidobacteria are abundant and active in rhizosphere soils metabolically [47,48]. A previous study indicated that Actinobacteria, Acidobacteria, and Proteobacteria form the dominant phyla in boreal forests [49,50]. Sequencing illustrated that the majority of the sequences identified were those of *Enterococcus*, the most enriched genus in the T6-1 rhizosphere and was also strongly present in the CK rhizosphere. As expected, the fraction of *Bacillus* was higher in T6-1 relative to that in the CK rhizosphere. It is presumed that the application of *Bacillus* T6-1 improved the rhizosphere bacterial community structure. The reduction in *Rhizoctonia* in the T6-1 rhizosphere indicated the antagonistic ability of T6-1 mentioned earlier. This is consistent with previous research, in which *B. subtilis* RB14-C was used as a biocontrol agent against *R. solani* [51].

In addition, the majority of the fungal sequences were assigned to Ascomycota, the most enriched phylum in the study samples. Previous studies have revealed that Ascomycota are ubiquitous and ecologically important in soils [52] and are the dominant fungi in continuous cropping soils [53]. The fraction of *Rhizoctonia* was remarkably lower in the T6-1 relative to that in the CK rhizosphere, illustrating that *Rhizoctonia* might entail pathogens that account for continuous cropping obstacles in poplar cultivation. As an important root pathogenic fungus, *R. solani* has since long been studied in various crops [54,55,56,57]. *Rhizoctonia solani* is a major root pathogen in different kinds of crops and may help develop natural disease suppression under continuous cropping [58].

We found that the application of *B. subtilis* T6-1 zymotic fluid can promote the production of poplars, inhibit pathogenic fungi in the soil, and improve the rhizosphere microbial community structure. Similar research has documented that *B. subtilis* serves as an environmentally compatible plant-protective agent in soils. For example, *B. subtilis* B068150 has been applied as a biocontrol agent against *Fusarium oxysporum* f. sp. *cucumerinum* [59,60].

## 5. Conclusions

In summary, we established that *B. subtilis* T6-1 could dramatically alleviate the limiting factors for the continuous cropping of poplar. It enhanced the structure of rhizosphere microbe communities, increased tree growth, and inhibited pathogenic fungi in continuously cropped poplars. Thus, T6-1 may potentially be useful to protect the strain against continuous cropping limiting factors for poplar cultivation.

## Figures and Tables

**Table 1:** Changes in DBH and plant height of poplar trees.

Treatment	DBH (mm) in 2018/10	DBH (mm) in 2020/10	Plant Height (m) in 2018/10	Plant Height (m) in 2020/10
T6-1	38.54 ± 3.88 ^a^	124.16 ± 9.06 ^a^	4.15 ± 0.36 ^a^	11.34 ± 0.62 ^a^
CK	35.23 ± 4.12 ^b^	111.13 ± 8.84 ^b^	3.99 ± 0.30 ^a^	10.73 ± 0.48 ^b^

Data are given as the mean ± SE. Different lowercase superscript letters in the same column designate a remarkable difference at *p* < 0.05. “T6-1”, poplar trees exposed to bacterial zymotic fluid of T6-1; “CK”, poplar trees exposed to equal volume of sterile zymotic fluid.

**Table 2:** Edaphic properties of soils in March 2018 and October 2020.

		OC (g/kg)	AP (mg/kg)	AK (mg/kg)	TN (mg/kg)	pH
Mar-13	T6-1	6.12 ± 0.08 ^c^	35.91 ± 4.24 ^b^	132.06 ± 2.38 ^c^	758.98 ± 9.26 ^b^	7.19 ± 0.02 ^b^
	CK	6.05 ± 0.07 ^c^	35.63 ± 2.33 ^b^	132.28 ± 1.44 ^c^	765.82 ± 7.43 ^b^	7.23 ± 0.02 ^b^
Oct-15	T6-1	7.12 ± 0.03 ^a^	46.72 ± 3.02 ^a^	231.79 ± 4.23 ^a^	1092.49 ± 44.79 ^a^	7.39 ± 0.02 ^a^
	CK	6.27 ± 0.04 ^b^	35.38 ± 2.77 ^b^	139.20 ± 2.03 ^b^	791.58 ± 3.77 ^b^	7.07 ± 0.04 ^c^

Data are given as the mean ± SE. Different lowercase superscript letters in the same column designate a remarkable difference at *p* < 0.05. “Mar-13” is before treatments and “Oct-15” is after treatments with zymotic fluid and sterile zymotic fluid; “T6-1”, poplar trees exposed to bacterial zymotic fluid of T6-1, “CK”, poplar trees exposed to an equal volume of sterile zymotic fluid.

**Table 3:** Quantity of microorganisms in rhizosphere soils of the two treatments.

Treatment	Culturable Microbial Contents after the Two Treatments		Total Microbial Contents after the Two Treatments	
	Bacterial × 10^7^ (cfu/g Soil)	Fungal × 10^6^ (cfu/g Soil)	Bacterial × 10^7^ (Copies/μL)	Fungal × 10^4^ (Copies/μL)
T6-1	9.71 ± 0.12 ^a^	5.00 ± 0.25 ^b^	1.17 ± 0.11 ^a^	2.19 ± 0.01 ^b^
CK	0.96 ± 0.17 ^b^	9.25 ± 0.49 ^a^	0.71 ± 0.02 ^b^	2.95 ± 0.10 ^a^

Data are given as the mean ± SE. Different lowercase superscript letters in the same column designate a remarkable difference at *p* < 0.05. “T6-1”, poplar trees exposed to bacterial zymotic fluid of T6-1; “CK”, poplar trees exposed to an equal volume of sterile zymotic fluid.

**Table 4:** Diversity along with richness indices of bacterial, as well as fungal communities from the two soil treatments.

	Sample	Cutoff	OTUs	ACE	Chao	Shannon	Simpson	Coverage
Bacterial	T6-1	0.03	2161 ± 44.84 ^a^	2362.75 ± 68.25 ^a^	2362.00 ± 102.20 ^a^	6.06 ± 0.23 ^a^	0.023 ± 0.013 ^a^	0.980169
	CK	0.03	2106 ± 106.71 ^a^	2384.41 ± 82.6 ^a^	2402.16 ± 78.39 ^a^	6.20 ± 0.12 ^a^	0.012 ± 0.004 ^b^	0.980821
Fungal	T6-1	0.03	829.33 ± 96.45 ^a^	923.56 ± 90.50 ^b^	919.39 ± 93.32 ^b^	4.08 ± 0.02 ^b^	0.045 ± 0.007 ^a^	0.9967853
	CK	0.03	901 ± 7.55 ^a^	1032.86 ± 57.48 ^a^	1022.94 ± 93.32 ^a^	4.34 ± 0.02 ^a^	0.040 ± 0.005 ^a^	0.996280

Data are given as the mean ± SE. Different lowercase superscript letters in the same column designate a remarkable difference at *p* < 0.05. “T6-1” poplar trees exposed to bacterial zymotic fluid of T6-1; “CK”, poplar trees exposed to an equal volume of sterile zymotic fluid.

## Data Availability

Most data are presented in the article and SRA database (Bioproject accession number: PRJNA835433). The raw data are presented in Supplementary Materials. The other materials can be provided upon request.

## Supplementary Materials

The following are available online at https://www.mdpi.com/article/10.3390/biology11050791/s1, Figure S1: NMDS plots on OTU level showing the distribution patterns of (a) bacteria and (b) fung. Abundance data were transformed for equal sampling depth prior to generation of the distance matrix used for the non-metric multidimensional scaling (NMDS) plot.
