# Supplementary material for: Effects of Bacillus subtilis T6-1 on the Rhizosphere Microbial Community Structure of Continuous Cropping Poplar
Source: Biology (Basel). 2022 May 23;11(5):791. doi: 10.3390/biology11050791 (PMC9138279; doi:10.3390/biology11050791)
Supplement: Supplementary file 1 [file biology-11-00791-s001.zip › biology-1675884-supplementary.pdf]

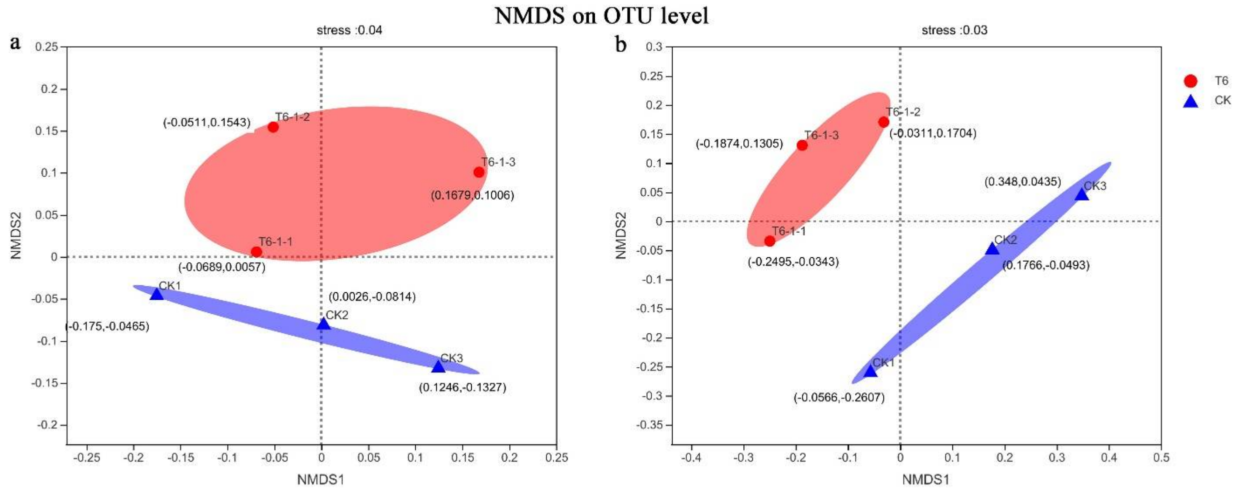

Figure S1. NMDS plots on OTU level showing the distribution patterns of (**a**) bacteria and (**b**) fung. Abundance data were transformed for equal sampling depth prior to generation of the distance matrix used for the non-metric multidimensional scaling (NMDS) plot. The Bray–Curtis index was used as the distance matrix. Stress < 0.05 showing good representativeness. The distance matrix of Bray-Curtis:$D_{Bray-Curtis} = 1 - 2\frac{\sum \min(S_{A,i},S_{B,i})}{\sum S_{A,i}+\sum S_{B,i}}$. $S_{A,i}$ means the number of sequences contained in the *i*th taxon in sample A, $S_{B,I}$ means the number of sequences contained in the *i*th taxon in sample B.
